# Patient acceptability of CITOBOT for cervical cancer screening: A mixed-method study

**DOI:** 10.1371/journal.pone.0325805

**Published:** 2025-06-24

**Authors:** Marcela Arrivillaga, Maria Del Mar Torres, Daniela Neira, Juan Pablo García-Cifuentes, Hernán Dario Vargas-Cardona, Mérida Rodríguez-López, Paula C. Bermúdez

**Affiliations:** 1 Department of Public Health and Epidemiology, Pontificia Universidad Javeriana Cali, Cali, Colombia; 2 Department of Maternal and Child Health, Pontificia Universidad Javeriana Cali, Cali, Colombia; 3 Red de Salud Ladera ESE, Alcaldía de Cali, Cali, Colombia; 4 Department of Electronics and Computer Sciences. Pontificia Universidad Javeriana Cali, Cali, Colombia; 5 Faculty of Health Sciences, Universidad Icesi & Centro de Investigaciones Clínicas Fundación Valle del Lili, Cali, Colombia; Teikyo University, School of Medicine, JAPAN

## Abstract

This study assessed the acceptability of CITOBOT, a device for early cervical cancer screening in a real-world pilot setting as part of a translational research project aimed at designing and clinically validating a portable, cost-effective device supported by artificial intelligence. The authors adopted the Theoretical Framework of Acceptability for its utility in evaluating patient acceptability within complex interventions’ development, piloting, and feasibility phases. We employed a mixed-method study, with 20 consecutive participants recruited from a specialized cancer healthcare center in Cali, Colombia. Data collection included a sociodemographic, gynecological-obstetric, behavioral survey, a validated patient acceptability scale, alongside open-ended interview questions. No adverse effects were reported seven days post-testing. The findings were promising, with all participants expressing high overall acceptability. Retrospective acceptability, focusing on the evaluation after device pilot testing, revealed that participants felt comfortable with the device, found it coherent with the purpose of early cervical cancer detection, and did not perceive the test as an additional burden compared to conventional cytology screening. Regarding prospective acceptability, which assesses anticipated acceptability before full implementation, three results stand out: i) All participants stated that they would intend to attend their health service if called for testing with CITOBOT; ii) they perceived opportunity costs, such as timely delivery of results, expedited diagnosis and treatment, and improved accessibility for women with limited resources or geographical barriers to healthcare access; and iii) participants viewed CITOBOT as highly effective in preventing cervical cancer deaths, indicating a strong belief in its potential to impact public health outcomes positively. Addressing concerns related to discomfort, inconvenience, and timely delivery of results, CITOBOT shows promise in enhancing cervical cancer screening participation and adherence, especially among underserved populations.

## Introduction

From the perspectives of healthcare services research and implementation science, the scalability and effectiveness of complex interventions depend significantly on patient acceptability [1–5]. In the context of cervical cancer, primary prevention through vaccination against human papillomavirus (HPV) has faced challenges, such as achieving broad coverage and overcoming barriers to access. Secondary prevention, which includes screening methods such as cytology and HPV DNA testing, has also encountered obstacles, particularly related to equitable access to screening services [6]. These challenges in prevention efforts have highlighted the need for innovations in early detection strategies. A major obstacle remains the timely delivery of results to prevent the risk of disease progression, which in recent years has driven the development of new devices aimed not only at improving the timeliness of screening but also at reducing the burden of multiple clinical visits through more efficient and accurate diagnostic processes [7].

The Pocket Colposcope [8], Veda-scope [9,10], Evalyn brush® [11], and Viba-brush® [9], among other devices [10,12,13], have been described in the published literature as innovative tools for cervical cancer screening [14]. However, patient acceptability regarding their use, particularly for devices similar to those used in Pap smear or VIA-VILI tests, has not been thoroughly explored. Sekhon and colleagues (2017) noted that while many healthcare interventions claim to have evaluated acceptability, there remains a notable gap in robust research in this domain. According to these authors, acceptability is a multifaceted concept encompassing seven components: affective attitude, burden, perceived effectiveness, ethics, intervention coherence, opportunity costs, and self-efficacy [12].

Women diagnosed with pre-cancerous lesions or gynecological cancers often endure significant reductions in their quality of life [15,16]. Moreover, the acceptability of devices and procedures employed in screening, diagnosis, treatment, and rehabilitation services remains largely unexplored. On a preventive front, inadequate equipment, insufficiently trained medical staff, and resource constraints can impede the execution of screening and diagnostic procedures. This challenge is especially pronounced in rural and underserved areas across diverse regions worldwide, where access to screening tests may be severely limited or absent [17,18].

This study is part of a translational research initiative aimed at developing and clinically validating CITOBOT, a device for early cervical cancer screening. CITOBOT [19] consists of a disposable transvaginal component with bidirectional opening capabilities, adjustable in both height and angle to optimize usability and patient comfort. It is equipped with a removable endoscopic camera featuring an integrated LED lighting unit for capturing high-quality cervical images, which connects seamlessly to a real-time display unit. The system includes an advanced image analysis module powered by artificial intelligence algorithms, embedded in a mobile application that functions offline, eliminating the need for continuous internet access. Additionally, CITOBOT incorporates software available on mobile and web platforms, which guides healthcare providers step by step through the screening process. This software transmits cervical images to the AI module, delivers a risk assessment for cervical cancer in less than five seconds, and enables providers to access patient screening histories. Moreover, the system supports retraining of the AI module, ensuring continuous improvement in diagnostic performance. Currently, CITOBOT has two patents pending: 1) Utility Model Patent in Colombia (NC2024/0008763, filed on June 30, 2024) and 2) PCT (International Application No. PCT/IB2024/057963, filed on August 16, 2024).

After completing the initial phases, which involved designing both hardware and software components, we assessed the acceptability of CITOBOT from the participants’ perspective in a real-world pilot setting during the patient translation phase. This evaluation aimed to refine the device, increasing its potential adoption in cancer preventive services. Consequently, this paper aims to present the findings obtained from this stage of evaluating the device’s acceptability.

## Materials and methods

### Study design

A mixed-method study [20] was conducted based on the Theoretical Framework of Acceptability (TFA) (v2) proposed by Sekhon et al., which is relevant to the entire complex intervention development and evaluation cycle. Therefore, in the pilot testing phase, before proceeding to large-scale device testing, we assess its acceptability among participants. The integration of quantitative and qualitative approaches allows for a comprehensive understanding of the individual perceptions of women regarding the device. Acceptability is comprehended as a *multifaceted construct that reflects the extent to which people delivering or receiving a healthcare intervention consider it to be appropriate, based on anticipated or experienced cognitive and emotional responses to the intervention* (p. 4) [12]. The project was reviewed and approved by the Ethics Committee for Research in Human Health of the ‘Pontificia Universidad Javeriana Cali’ (#002/2022) and by ‘Unicáncer’ in Cali, Colombia (04/06/2021). Written informed consent was obtained from all participants prior to their inclusion in the study, ensuring that they understood the purpose of the research, the procedures involved, and their right to withdraw at any time without repercussions.

### Participants, recruitment, and procedures

The study includes consecutive participants recruited at a specialized cancer healthcare center in Cali, Colombia, between February and April 2024. Study information was disseminated via email, social media announcements, and in-person presentations by the research team during monthly meetings. Eligibility criteria included women aged 21–59 years with a present cervix and a documented history of previous cytological screening. To ensure diverse representation, participants were intentionally selected based on factors known to influence cervical anatomy and morphology, such as nulliparity, multiparity, menopausal status, and history of cervical procedures like cauterization or conization. These factors can impact cervical size, shape, and appearance due to variations in age, parity, hormonal cycling, and prior medical interventions. Exclusion criteria encompassed self-reported allergies to acetic acid or Lugol’s solution, recent sexual intercourse, vaginal douching, use of vaginal medications or spermicides within 2 days before testing, menstruation, pregnancy, and postpartum period less than 6 weeks. After the medical record review, additional exclusions included the history of cervical cancer, coagulation disorders or anticoagulant use, recurrent miscarriages, bleeding during prior colposcopy procedures, and physical or mental disabilities that could impair study participation. Participants could withdraw if intraoperative bleeding occurred or at their voluntary request.

The trials with CITOBOT were conducted in a medical office setting by a gynecology specialist previously trained on a GYN/AID Gaumard S503-4 gynecological simulator at a Simulated Hospital facility. Data collection employed a mixed-method tool to assess patient acceptability (S1 File) comprising: i) a survey assessing sociodemographic, gynecological-obstetric, and behavioral factors potentially associated with device acceptability through clinical or behavioral inference, ii) a patient acceptability scale developed and validated by the authors via expert consultation and pilot testing, and iii) an open-ended interview guide. All participants attended a 7-day post-procedure follow-up appointment, during which no adverse events were reported.

Quantitative data was collected using a scale developed by the authors, with scores ranging from 0 to 12 points. The scale assessed six factors through individual Likert-type items rated from 0 (low acceptability) to 2 (high acceptability). Factors were ‘overall device perception’, ‘comfort’, ‘pain’, ‘safety’, ‘material’, and ‘comparison to a traditional speculum’. A total sum score was calculated across all items, with higher scores indicating greater acceptability. For analysis, total scores were categorized into three acceptability parameters: low (0–3 points), moderate (4–8 points), and high (9–12 points).

Qualitative data were gathered via semi-structured interviews guided by the Theoretical Framework of Acceptability (TFA v2). The interview guide explored the seven TFA constructs: affective attitude, burden, perceived effectiveness, ethical considerations, intervention coherence, opportunity costs, and self-efficacy. Participants were also asked to provide feedback on possible improvements to the CITOBOT device.

### Data analysis

Descriptive statistics were used to summarize the sociodemographic, gynecological-obstetric, and behavioral characteristics of the cases. Quantitative data from the patient acceptability scale were summarized using frequencies and percentages for categorical variables corresponding to each scale item. Additionally, total scores were calculated for individual participants. Qualitative data were obtained through semi-structured interviews, audio-recorded, and professionally transcribed verbatim. A thematic analysis approach was employed, guided by the constructs outlined in the Theoretical Framework of Acceptability (TFA v2). Identified themes were categorized as relating to either retrospective or prospective acceptability factors and further delineated through color coding. Relevant quotations exemplifying each TFA construct were selected for inclusion.

To enhance validity, triangulation of qualitative themes and quantitative data was conducted to identify and display convergence [20] regarding factors influencing the acceptability of the tested cervical screening device. In reporting results, verbatim quotations from participants are presented with numerical identifiers, age, and relevant obstetrical/gynecological characteristics (e.g., nulliparity, multiparity, menopausal status, history of cervical procedures) to provide context.

## Results and discussion

### Participants profile

Twenty women participated in the study, comprising 20% nulliparous, 45% multiparous, 25% menopausal, and 10% with a history of previous cervical procedures. Most participants were from lower socioeconomic backgrounds (55%), which could impact access to healthcare and preventive measures. Behavioral trends revealed low condom use (75%) and a lack of active sexual life (65%). Although 65% had undergone HPV testing within the past five years, 35% had never been tested, highlighting a gap in screening. Additionally, the majority had experienced multiple pregnancies (65%) and vaginal births (90%), emphasizing the importance of reproductive health considerations in this population. (Table 1).

**Table 1:** Sociodemographic, gynecological-obstetric, and behavioral characteristics of participants in the CITOBOT study (n = 20), Cali, Colombia, 2024.

		n	%
**Sociodemographic Characteristics**			
**Age**	Aged 21–25 years	3	15%
	Aged 26–45 years	10	50%
	Aged over 45 years	7	35%
**Educational level**	Elementary School	6	30%
	High school	8	40%
	Vocational or Technical	4	20%
	Undergraduate Degree	2	10%
**Marital status**	Single	5	25%
	Married or Cohabitating	13	65%
	Separated or Widowed	2	10%
**Occupation**	Employed	11	55%
	Self-employed or Independent Worker	3	15%
	Homemaker	5	25%
	Student	1	5%
**Socioeconomic strata** ^a^	Low (1 y 2)	11	55%
	Medium (3 y 4)	6	30%
	High (5–6)	3	15%
**Gyneco-Obstetric and Behavioral Characteristics**			
**Pregnancies**	0	4	20%
	1–3	13	65%
	4 or more	3	15%
**Number of vaginal births**	1–3	18	90%
	Four or more	2	10%
**Abortions**	Yes	4	20%
	No	16	80%
**Active sexual life**	Yes	7	35%
	No	13	65%
**Condom use**	Yes	5	25%
	No	15	75%
**HPV Test**	Yes, within the last five years	13	65%
	Yes, more than five years ago	0	0%
	No	7	35%
**HPV Test Result**	Negative	8	40%
	Positive	4	20%
	Don’t know/No response	8	40%

^a^In Colombia, socioeconomic strata (´estratos socioeconómicos´) are a classification system used to categorize residential areas based on their physical and economic characteristics. These strata range from 1 (low-low) to 6 (high), with the purpose of implementing utility subsidies and taxes. Lower strata (1 and 2) receive subsidies, middle strata (3 and 4) pay standard rates, and higher strata (5 and 6) contribute additional fees to subsidize lower-income groups. This classification is widely used in public policy and research to understand socioeconomic disparities.

### Participants acceptability

The total score on the acceptability scale indicated that 75% of the participants had high acceptability, while 25% showed moderate acceptability of CITOBOT; none scored low acceptability.

**Fig 1:**
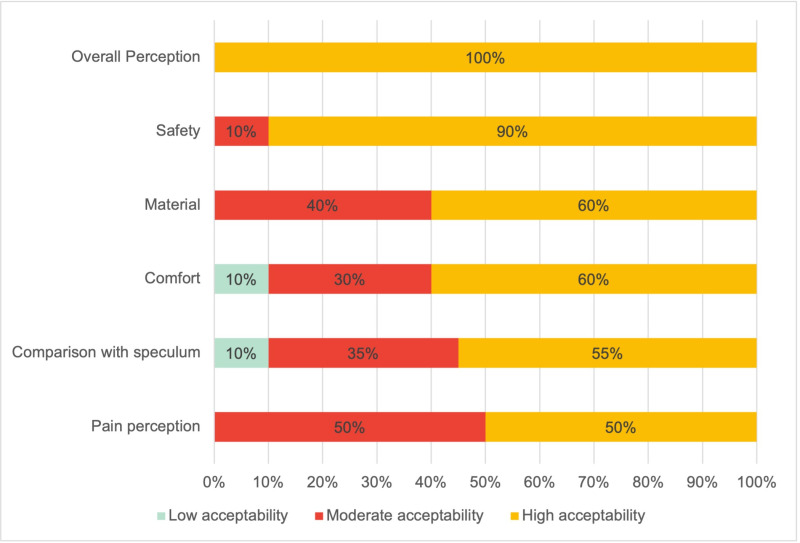
Acceptability by factors among participants who tested with CITOBOT for early detection of cervical cancer (n = 20), Cali, Colombia, 2024.

Results concerning each factor evaluated with the scale indicate that all participants reported an overall positive experience with CITOBOT, 90% felt secure during the test, 60% expressed no discomfort with the material, and 40% experienced some discomfort. Pain perception ranged from no pain, reported by 50%, to some pain, reported by another 50%. Lastly, compared to the traditional speculum, 55% found CITOBOT device better, 35% found it equivalent, and 10% found it worse, a percentage matching those who reported the device to be very uncomfortable (Fig 1).

**Fig 2:**
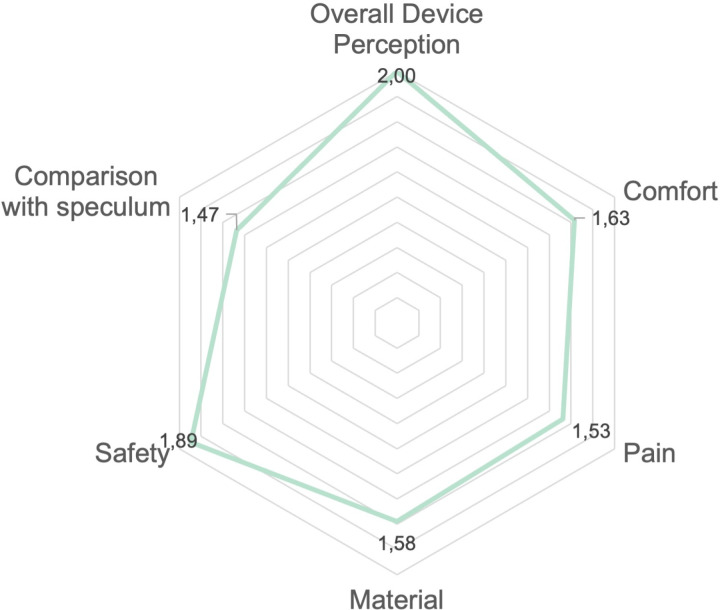
Average scores per factor from 0 (low) to 2 (high) acceptability for CITOBOT device (n = 20), Cali, Colombia, 2024.

The radar chart displayed in Fig 2 illustrates participants’ perceptions of the CITOBOT device across six key factors: overall device perception, comfort, pain, material, safety, and comparison with the speculum. Each axis represents one factor, with scores ranging from 0 (negative perception) to 2 (positive perception). The chart visually compares the device’s performance across these attributes, highlighting both strengths and areas for improvement. The highest mean rating was observed for the ‘overall perception’ factor, followed by ‘safety’ perception, indicating strong positive feedback in these areas. In contrast, ‘comparison with the speculum’ received the lowest mean score, suggesting potential areas for enhancement. These scores, based on participant feedback and averaged for each factor, provide a comprehensive overview of the device’s acceptability.

### Retrospective and prospective acceptability

Retrospective acceptability refers to evaluating the acceptability of the device after its pilot testing in the simulated environment. Participants provided feedback on how CITOBOT device was perceived and how this could influence its future adoption and use. The findings show that participants felt comfortable with the device, except those with previous cervical procedures (affective attitude); for all women, the device was coherent with its purpose of early cervical cancer detection (intervention coherence); and the test did not represent an additional burden in terms of time or effort compared to what is usually experienced when attending cytology screening (burden).

On the other hand, the results regarding prospective acceptability, understood as the anticipated evaluation of the acceptability of the device before its full implementation in actual practice, showed that participants considered the device would be appropriate and accepted by women in general (ethicality), that it would have benefits such as timely delivery of results, expediting diagnosis and treatment, which would be especially favorable for women with limited resources who face greater barriers to accessing health centers due to geographic or economic reasons (opportunity costs); that it would be highly effective in preventing deaths from cervical cancer (perceived effectiveness); that they would intend to attend a screening appointment if CITOBOT were used, and that they would recommend it to other women (self-efficacy/intention). Table 2 presents the convergent data for each construct from the Theoretical Framework of Acceptability (TFA v2), integrating the most significant quotes and the level of patient acceptability.

**Table 2:** Convergent data on the acceptability of CITOBOT based on constructs from the Theoretical Framework of Acceptability (TFA v2), Sekhon et al. (2017). Cali, Colombia, 2024.

Acceptability Construct	Quotes	Patient Acceptability Level
**Retrospective Acceptability**		
**Affective Attitude:** *How did you feel during the test with CITOBOT?*	*‘I felt comfortable, secure, it was a different experience because normally it’s annoying with the speculum, but here it didn’t feel that way.’ (29 years old, multiparous)*	High
	*‘It was fine, I didn’t feel any discomfort whereas, with the Pap smear, it’s uncomfortable because they pinch and I bleed.’ (59 years old, menopausal)*	High
	*‘I felt super good! Comfortable.’ (33 years old, multiparous)*	High
	*‘I felt good, a little uncomfortable. I needed to relax and be calm.’ (26 years old, nulliparous)*	Moderated
	*‘Fine, but uncomfortable due to the atrophy diagnosed by my doctor. ‘ (55 years old, menopausal)*	Moderated
**Intervention Coherence:** *Was the purpose of the test with CITOBOT clear to you?*	*‘The test was to see if the device is good, comfortable, and safe, and in the future, help prevent cervical cancer.’ (21 years old, multiparous)*	High
	*‘It will help women reduce the risk of cancer.’ (33 years old, multiparous)*	High
	*‘I understood the purpose of the test, about its comfort, and in the future, the impact it will have on women’s health.’ (43 years old, multiparous)*	Moderated
**Burden:** *Did the test with CITOBOT require a significant amount of additional time or effort?*	*‘No, after everything I’ve been through! This was good.’ (54 years old, Post-conization)*	High
	*‘The test time was ideal.’ (50 years old, multiparous)*	High
	*‘...it seemed too fast to me, didn’t require much effort, and is very practical.’ (26 years old, nulliparous)*	Moderated
**Prospective Acceptability**		
**Ethicality:** *Do you think the test with CITOBOT would be an appropriate and acceptable procedure for women in general?*	*‘I do believe so, they give quick results. Waiting for results makes you anxious.’ (54 years old, Post-conization)*	High
	*‘Yes, it’s more comfortable, innovative, and good for women who will have a quicker diagnosis.’ (21 years old, multiparous)*	High
	*‘Yes, effective for women with limited resources who have to go to the health center repeatedly to get one result after another.’ (31 years old, nulliparous)*	High
**Opportunity costs:** *What benefits do you think CITOBOT could have in preventing cervical cancer?*	*‘Yes, especially for women who are afraid. It’s better to prevent.’ (54 years old, Post-conization)*	High
	*‘It will have benefits for preventing cervical cancer because the risk will be detected faster, and diagnosis and treatment would be quicker. Fewer women might die, especially if it’s taken to places with limited resources.’ (35 years old, nulliparous)*	Moderated
	*‘Yes, because it will be faster and it won’t be too late where nothing can be done anymore.’ (45 years old, multiparous)*	Moderated
**Perceived Effectiveness:** *Do you think CITOBOT could help reduce the risk of women developing or dying from cervical cancer?*	*‘Yes, my grandmother died of cervical cancer, and it’s very important to get checked.’ (33 years old, multiparous)*	High
	*‘I think it will help women not die from cancer because they won’t be scared of the test anymore.’ (24 years old, multiparous)*	High
	*Well, of course, because everything will be more effective.’ (43 years old, multiparous)*	High
**Self-Efficacy (Intention):** *If your healthcare provider scheduled you for a test with CITOBOT, would you attend?*	*‘Yes, I would have liked to be diagnosed that way and avoided my procedure.’ (24 years old, Post-conization)*	High
	*‘Yes, and I would recommend it.’ (50 years old, multiparous)*	High
	*‘Absolutely.’ (45 years old, multiparous)*	Moderated

This study aimed to evaluate the acceptability of CITOBOT, a device for cervical cancer screening among volunteer participants using a mixed-method approach. We adopted the Theoretical Framework of Acceptability (TFA v2), proposed by Sekhon and colleagues in 2017, as a reference framework due to its utility in evaluating patient acceptability within the development, piloting, and feasibility phases of complex interventions, as described in the guidelines of the Medical Research Council [4,5]. The findings provide valuable insights into patient perspectives that can inform the development and implementation of this innovative screening tool.

A key strength of this study lies in its patient-centered design, which aligns with the growing recognition that patient acceptability is a critical determinant of the scalability and effectiveness of complex healthcare interventions [21]. By directly engaging participants and soliciting their feedback, the study enhances the potential adoption and sustained use of CITOBOT within cervical cancer preventive services.

Several devices have been developed to improve cervical cancer screening protocols, aiming to increase participation rates and reduce the incidence of cervical cancer [14,22]. The emergence of portable devices could streamline the execution of additional cervical screening initiatives, particularly in under-resourced rural regions. These devices cater to individuals with limited proficiency in smear collection while assisting highly skilled cytologists [14]. CITOBOT, evaluated in this study shares similarities with those belonging to the category of Pap smear tests and VIA-VILI.

As previous studies have suggested, the likelihood that women will use a cervical cancer screening service depends on many factors, including attitudes and perceptions about cervical cancer itself, early detection, diagnosis, and treatment [23]. The findings of our study are very promising, with quantitative results that revealed high overall acceptability, with 75% of participants expressing high acceptability and the remaining 25% reporting moderate acceptability. None of the participants scored in the low acceptability range, indicating a generally positive reception of CITOBOT.

The qualitative data provided insights into the specific factors influencing patient acceptability. Retrospective acceptability, which focuses on the evaluation after device pilot testing, revealed that participants felt comfortable with CITOBOT, found it coherent with the purpose of early cervical cancer detection, and did not perceive the test as an additional burden compared to conventional cytology screening. This feedback suggests that the device addresses common concerns and barriers associated with traditional screening methods.

In our study, only women with prior cervical procedures, such as conizations or cauterizations, or women who have not had sexual intercourse reported discomfort with CITOBOT. No woman reported pain during the test, while previous data reported in the literature shows that speculum insertion is one of the most disturbing and painful procedures due to its intrusive nature, and approximately 35% of women experience pain during a vaginal examination with a vaginal speculum [24,25]. It has also been recommended that physicians perform maneuvers to minimize pain and provide greater patient comfort [25–31]. CITOBOT has the attribute of its design that does not require the physician to perform rotations within the vaginal cavity to obtain a complete visual of the cervix. This factor may be related to the high and moderate acceptability results obtained regarding pain perception and favorability toward the manufacturing material.

Regarding prospective acceptability, which assesses anticipated acceptability before full implementation, three results stand out: i) All participants stated that they would intend to attend their health service if called for testing with CITOBOT; ii) they perceive opportunity costs, such as timely delivery of results, expedited diagnosis and treatment, and improved accessibility for women with limited resources or geographical barriers to healthcare access; and iii) importantly, participants viewed CITOBOT as highly effective in preventing cervical cancer deaths, indicating a strong belief in its potential to impact public health outcomes positively.

Methodologically, this study adds value by providing a content-valid scale for evaluating the acceptability of a medical device, as well as the convergent integration of results with the TFA constructs. While the number of references in the literature on patient acceptability has been increasing, it is necessary to generate more evidence on how patient acceptability can be evaluated quantitatively and qualitatively and offer specific materials for its operationalization [3], as outlined in this article.

Although the study results show promise, it is important to acknowledge certain limitations. The sample size was relatively small, and participants were recruited from a specialized cancer healthcare center, which may not fully represent the broader population’s diverse socioeconomic and cultural backgrounds. Selection criteria for participants could be adjusted based on variables such as pregnancy, postpartum status within six weeks, dyspareunia, vaginitis, vulvar pain or lesions, undergoing a procedure, or never having had vaginal intercourse. Women who might have an altered perception of pain during speculum insertion should also be excluded [32,33]. Additionally, as the study focused on patient acceptability, further research is needed to evaluate the acceptability of CITOBOT among healthcare providers, who play a crucial role in its successful implementation. For example, to evaluate the acceptability of the device by physicians so that it can be adopted in clinical practice and effectively implemented in cervical cancer preventive services, aspects such as ease of use, compatibility with current clinical practice, perceived clinical benefits, safety, reliability, cost-effectiveness, training, and support could be considered. This is important because the device’s complexity may affect its acceptability. It should integrate easily into existing procedures and workflows in clinical practice, and physicians may be more inclined to use a device if they see clear benefits for their participants.

Future research could explore the acceptability of CITOBOT across different geographical regions, socioeconomic status, and cultural contexts. Longitudinal studies could also assess the long-term sustainability of patient acceptability and adherence to screening programs. Furthermore, investigating the perspectives of healthcare providers, policymakers, and other stakeholders would provide a comprehensive understanding of the factors influencing the device’s widespread adoption and integration into existing healthcare systems.

## Conclusions

In conclusion, this study offers promising evidence of patient acceptability of CITOBOT for cervical cancer screening, aligning with the growing emphasis on patient-centered care and the recognition that acceptability is a critical determinant of intervention effectiveness. By addressing concerns related to discomfort, burden, and timely delivery of results, CITOBOT can potentially improve cervical cancer screening uptake and adherence, particularly among underserved populations. However, continued research and stakeholder engagement is necessary to successfully translate and implement this innovative technology into real-world settings.

## Supporting information

S1 File A mixed-methods tool to assess patient acceptability of the CITOBOT device for cervical cancer screening. Legend: This file contains the mixed-methods tool used to evaluate patient acceptability of the CITOBOT device for cervical cancer screening. It includes both qualitative and quantitative components designed to assess key dimensions of acceptability.
(DOCX)
