# Supplementary material for: Patient acceptability of CITOBOT for cervical cancer screening: A mixed-method study
Source: PLoS One. 2025 Jun 24;20(6):e0325805. doi: 10.1371/journal.pone.0325805 (PMC12186933; doi:10.1371/journal.pone.0325805)
Supplement: S1 File — A mixed-methods tool to assess patient acceptability of the CITOBOT device for cervical cancer screening. Legend: This file contains the mixed-methods tool used to evaluate patient acceptability of the CITOBOT device for cervical cancer screening. It includes both qualitative and quantitative components designed to assess key dimensions of acceptability. (DOCX) [file pone.0325805.s001.docx]

**Supplement 1. A mixed-methods tool to assess patient acceptability of the CITOBOT device for cervical cancer screening**

**Study Patient Code** |___|___|

1. **Sociodemographic Factors**
2. ¿How many years old are you? |___|___| years old
3. ¿What is or was the highest level of education you have attained?
   1. I__I Elementary School
   2. I__I High school
   3. I__I Vocational or Technical
   4. I__I Undergraduate Degree
   5. I__I None
   6. I__I No response
4. ¿What is your current marital status?
   1. I__I Single
   2. I__I Married or Cohabitating
   3. I__I Separated or Widowed
   4. I__I No response
5. ¿What is your current employment status or occupation?"
6. I__I Employed
7. I__I Self-employed or Independent Worker
8. I__I Homemaker
9. I__I Student
10. I__I No response
11. According to your utility bill, what is the socioeconomic stratum of your household?
12. I__I Low (1 y 2)
13. I__I Medium (3 y 4)
14. I__I High (5-6)
15. I__I Don't know/No response
16. **Gyneco-obstetric and behavioral factors**
17. ¿How many pregnancies have you had? |___|___|
18. ¿How many normal deliveries have you had? |___|___|
19. ¿How many abortions have you had? |___|___|
20. ¿Do you have an active sex life at the moment?
21. I__I YES
22. I__I NO
23. ¿During sexual intercourse, do you or your partner use condoms?
24. I__I Always
25. I__I Frequently
26. I__I Sometimes
27. I__I Never
28. ¿Have you undergone HPV DNA testing?
29. I__I Yes, less than 5 years ago
30. I__I Yes, more than 5 years ago
31. I__I No
32. I__I Don't know/No response
33. ¿Have you undergone any procedures on the uterine cervix?
34. I__I Yes ➔ __________________________

How long ago? |___|___| months |___|___| years

1. I__I No
2. I__I Don't know/No response
3. **Patient Acceptability Scale (0-12)**
4. ¿How was your overall experience with the new device?
5. I__I Poor
6. I__I Fair
7. I__I Good
8. ¿How did you feel about the comfort of the new device?
9. I__I Very uncomfortable
10. I__I Somewhat uncomfortable
11. I__I Comfortable
12. ¿How was the new device in terms of pain?
13. I__I Severe pain
14. I__I Some pain
15. I__I No pain
16. ¿How did you feel about the material of the new device?
17. I__I Very uncomfortable
18. I__I Somewhat uncomfortable
19. I__I No discomfort
20. ¿Did you feel safe during the procedure with the new device?
21. I__I Insecure
22. I__I Somewhat secure
23. I__I Secure
24. Compared to the traditional speculum, the new device was:
25. I__I Worse
26. I__I Equal
27. I__I Better

Total score: |___|___|

|  | **Low acceptability**  (0-3 points) | **Moderate acceptability**  (4-8 points) | **High acceptability**  (9-12 points) |
| --- | --- | --- | --- |
| Patient score |  |  |  |

1. **Open-ended interview questions based Theoretical Framework of Acceptability (TFA) (v2) proposed by Sekhon et al. (2017)**
   1. AFFECTIVE ATTITUDE: How did you feel during the test with CITOBOT? Please explain.

________________________________________________________________________________________________________________________________________________________________________________________________________________________________________________________________________________________________________

- 1. INTERVENTION COHERENCE: Was the purpose of the test with CITOBOT clear to you? Please explain.

________________________________________________________________________________________________________________________________________________________________________________________________________________________________________________________________________________________________________

- 1. BURDEN ASSOCIATED WITH PARTICIPATION IN THE INTERVENTION: Did the test with CITOBOT require a significant amount of additional time or effort? Please explain.

________________________________________________________________________________________________________________________________________________________________________________________________________________________________________________________________________________________________________

- 1. ETHICAL CONSIDERATIONS OF THE INTERVENTION: Do you think the test with CITOBOT would be an appropriate and acceptable procedure for women in general? Please explain.

________________________________________________________________________________________________________________________________________________________________________________________________________________________________________________________________________________________________________

- 1. OPPORTUNITY COSTS: What benefits do you think CITOBOT could have in preventing cervical cancer?

________________________________________________________________________________________________________________________________________________________________________________________________________________________________________________________________________________________________________

6. PERCEIVED EFFECTIVENESS: Do you think CITOBOT could help reduce the risk of women developing or dying from cervical cancer?

________________________________________________________________________________________________________________________________________________________________________________________________________________________________________________________________________________________________________

7. SELF-EFFICACY: If your healthcare provider scheduled you for a test with CITOBOT, would you attend?

________________________________________________________________________________________________________________________________________________________________________________________________________________________________________________________________________________________________________

8. Finally, what improvements do you suggest for the CITOBOT device?

________________________________________________________________________________________________________________________________________________________________________________________________________________________________________________________________________________________________________
